# Knockout of *GmCKX3* Enhances Soybean Seed Yield via Cytokinin-Mediated Cell Expansion and Lipid Accumulation

**DOI:** 10.3390/plants14142207

**Published:** 2025-07-16

**Authors:** Xia Li, Xueyan Qian, Fangfang Zhao, Lu Niu, Yan Zhang, Siping Han, Dongyun Hao, Ziqi Chen

**Affiliations:** 1 Key Laboratory of Molecular Cytogenetics and Genetic Breeding of Heilongjiang Province, College of Life Science and Technology, Harbin Normal University, Harbin 150025, China; 2 Institute of Agricultural Biotechnology, Jilin Provincial Key Laboratory of Agricultural Biotechnology, Jilin Academy of Agricultural Sciences (Northeast Innovation Center of Agricultural Science and Technology in China), Changchun 130033, China

**Keywords:** *GmCKX3*, cytokinin, seed size, yield, soybean

## Abstract

Soybean is a dual-purpose crop for food and oil, playing a crucial role in China’s grain production. Seed size and weight are key agronomic traits directly influencing the yield. Cytokinin oxidases/dehydrogenases (CKXs) specifically degrade certain isoforms of endogenous cytokinins (CKs), thereby modulating plant growth and seed development. However, their role in soybeans remains largely uncharacterized. In a previous genome-wide association study of 250 soybean core germplasms, we identified *GmCKX3* as a yield-related gene. To elucidate its function, we developed *GmCKX3*-deficient mutants using CRISPR/Cas9 gene editing in soybean Williams82 and conducted a three-year phenotypic analysis. Loss of *GmCKX3* function significantly enhanced the seed size and weight, which was attributed to an increased cell size and fat accumulation in the endosperm. This enhancement was driven by elevated endogenous CK levels resulting from suppressed *GmCKX3* expression. Subcellular localization revealed that *GmCKX3* resides in the endoplasmic reticulum and predominantly degrades the isopentenyladenine (iP)-type CK. Integrated transcriptomic and metabolomic analyses uncovered key genes and pathways involved in CK regulation, supporting *GmCKX3*’s central role in seed-trait modulation. These findings advance our understanding of cytokinin-mediated seed development and offer promising targets for molecular breeding aimed at improving the soybean yield.

## 1. Introduction

Soybean (*Glycine max* [L.] Merr.) is an essential global crop that provides vegetable oil and protein for both human consumption and livestock feed. Soybean occupies an important position in Chinese grain structure, highlighting the great importance of increasing the soybean yield and improving the seed quality [1]. The yield of soybeans is generally determined by the seed size and weight, of which the seed weight is usually positively associated with the seed size [2,3]. Despite the identification of numerous quantitative trait loci (QTL) related to seed size and shape, the actual functional genes responsible for these QTLs have seldom been pinpointed and applied in soybean breeding [4]. The effectiveness of molecular breeding in this crop has been documented to benefit from the clarification and validation of the functional genes associated with seed traits in soybeans [5]. Furthermore, there is still insufficient evidence specifically on the molecular regulatory networks controlling the seed traits in soybean compared to rice [6]. Therefore, in order to increase the soybean yield, there is a need to screen and discover the key genes controlling the seed size/weight and elucidate their molecular regulatory pathways [7].

Plant hormones have been confirmed to significantly regulate seed development, including seed size/weight, embryo development, endosperm development, and seed dormancy [7]. Cytokinins (CKs) are essential hormones involved in a series of developmental and physiological processes in plants, playing pivotal roles in regulating the proliferation and differentiation of plant cells. They have been recognized to promote shoot growth, inhibit root development, regulate fruit and seed development, postpone senescence, transfer nutritional signals, and respond to both abiotic and biotic stresses [8]. As a group of adenine derivatives, CKs are classified into isoprenoid and aromatic isoforms based on the different side chains at the *N*^6^ position, of which the isoprenoid CKs are more common and exist in higher quantities in plants than in non-plant organisms [9]. The isoprenoid CKs include *N*^6^-isopentenyladenine (iP), *trans*-zeatin (tZ), *cis*-zeatin (cZ), and dihydrozeatin (DZ), all of which are active forms of CKs that bind to the specific CK receptors [10]. CK homeostasis is sustained in vivo through biosynthesis, activation, degradation, and conjugation of the bioactive forms [9]. CKXs irreversibly cleave both the free-base and riboside forms of cytokinins at the *N*^6^-side chains to decrease the active cytokinin levels [11]. Different members of the *CKX* family exhibit varying affinities for distinct CK substrates. tZ and iP are readily catalyzed by CKXs from various plant species [12,13], whereas cZ is generally less susceptible [12]; since CKX recognizes the double bonds in the isoprenoid side chain, DZ and aromatic CKs are resistant to CKX [14]. Critically, CKXs are core determinants in modulating CK homeostasis with their phylogenetic characteristics as well as the unique biochemical and genetic properties [15,16,17]. CKXs are encoded by the *CKX* gene family, whose genetic abundance in both quantity and function promises its functional diversity among different plant species [18]. Previously, a great array of *CKXs* have been reported in various plants, including 7 *CKXs* in Arabidopsis (*AtCKX1~7*) [19], 11 *CKXs* in rice (*OsCKX1~11*) [20], 13 *CKXs* in maize (*ZmCKX1~12* and *ZmCKX4b*) [14], 11~14 *CKXs* in wheat [21,22,23,24], and 11 *CKXs* in barley (*HvCKX1~11*) [14]. However, comprehensive functional characterization of most of these CKXs remains incomplete.

The endogenous levels of CKs in plants are usually preserved by the functional modulations of *CKXs*, with one of the typical functions of *CKXs* being believed to affect yield-related traits in plants [25]. For example, Bartrina et al. revealed that a double mutation of *AtCKX3/AtCKX5* in Arabidopsis formed the enlarged inflorescences and floral meristems, thereby facilitating the production of more ovules [26]. This mutagenesis can promote the endogenous content of CKs, leading to a 55% increase in the grain yield. Conversely, an overexpression of *OsCKX2* in rice was discovered to decrease the grain number of transgenic rice, whereas *OsCKX2* suppression raised the level of CKs, eventually increasing the number of panicles and grains [20]. Similarly, there are also reports that, through the overexpression of *OsCKX2* in rice during grain development, elevated CK levels could boost the growth and maturation of the grains [27]. A natural variation in the wheat homologous gene *TaCKX6-D1* of *OsCKX2* was also reported to be significantly associated with the grain weight [28]. In addition, silencing *HvCKX1* and *HvCKX9* in barley using RNA interference stimulated the seed bank strength, resulting in an increased grain weight [29,30,31]. Furthermore, with the use of the full-length *CKX* sequences of Arabidopsis and rice as queries in a BLAST search, Nguyen et al. uncovered 17 putative *CKX* genes (*GmCKXs*) in a soybean genome, of which *GmCKX7-1* exhibited the highest expression level during seed filling in comparison to other developmental stages in a natural variant line [32]. Additionally, the variant line possessed a higher CK content than other lines and hence the promised yield performance. However, there is little data elucidating the detailed mechanism through which *GmCKXs* impact the yield-related traits by regulating CKs in soybean.

In a previous study, we conducted a genome-wide association study on a core germplasm collection of 250 soybean varieties from China, the United States, and Europe, with the identification of a yield-related gene, *GmCKX3* (*Glyma.17g054500*) [33]. However, there is still a lack of in-depth investigation into the mechanism by which this gene impacts the soybean yield. To further reveal the functionality of *GmCKX3* in regulating yield-related traits in soybeans, we prepared *cytokinin oxidase 3* (*Gmckx3*)-deficient mutants using CRISPR/Cas9 gene editing in soybean Williams82 and assessed their function. Using these soybean mutants, this study conducted a three-year phenotyping, coupled with cytological observation, physiological and biochemical assessments, as well as a combined analysis of transcriptomics and metabolomics, so as to elucidate the functional mechanism of *GmCKX3* in soybean grain size and weight. This study is anticipated to provide a theoretical basis for the functional mechanism of *GmCKX3* in regulating the seed traits of soybeans, thereby facilitating the evaluation of the application potential of the gene for high-yield soybean breeding.

## 2. Results

### 2.1. Preparation of GmCKX3-Genome-Edited Soybean

According to the information provided at Ensembl Plants (https://ngdc.cncb.ac.cn/soyomics/transcriptome/tissues, accessed on 12 December 2024), *GmCKX3* (ID# *Glyma.17g054500*) belongs to the FAD domain cytokinin oxidase/dehydrogenase protein gene family with a single transcript. It has a full length of 2184 bp with five exons, encoding a polypeptide of 535 amino acids of molecular weight 60.95 kDa.

**Figure 1:**
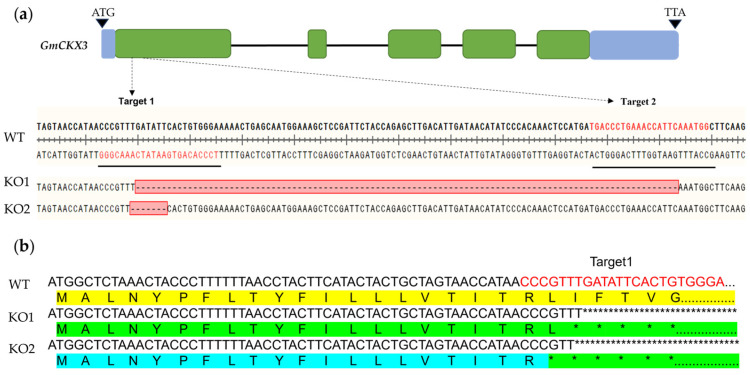
Preparation of gene-edited *GmCKX3* soybean. (**a**) The gene structure and sequence polymorphism of *GmCKX3*, as well as the sequencing validation of the gene-edited mutants. The structure of *GmCKX3* consists of five exons interrupted by four introns, and the location of the two-gRNA target is within the first exon of the cytokinin dehydrogenase domain. The green boxes indicate the exons, and the lines between the boxes indicate introns. The blue boxes indicate non-coding areas. The nucleotides beneath the exon 1 are the sgRNA target site sequences. The red-dashed lines represent the missing bases. (**b**) The diagram of amino acid deletion in gene-edited materials. Yellow, green, and blue represent amino acid sequences; asterisks represent missing amino acids, respectively; and red font represents the location of target 1.

In order to clarify the function of *GmCKX3*, we designed a dual-site base deletion to the first exon of *GmCKX3* at the 5′ end (Figure 1a) driven by an ATU6-26 promoter (Figure S1). The sgRNA was expressed and transformed into soybean Williams82. Through careful screening, two mutant soybean lines with free *Cas9* were produced with homozygous dual-target deletion, designated as KO1 (*Gmckx3-1*) and KO2 (*Gmckx3-2),* respectively, in T1 generation (Figure 1a). A sequence analysis revealed that KO1 and KO2 possessed the sequence knockout of 104 bp and 7bp at the first exon of *GmCKX3*, respectively (Figure 1a). These mutations led to a translational termination of *GmCKX3* (Figure 1b). The KO1 and KO2 mutations were stabilized in the T2 to T4 generations and further carried out for agronomy and functional identification.

### 2.2. Loss of Function GmCKX3 Increases Endogenous Cytokinin Levels in Soybean

**Figure 2:**
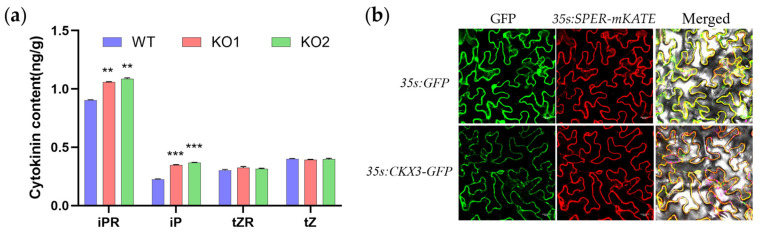
Loss of function: *GmCKX3* increases the endogenous cytokinin levels in soybeans. (**a**) The relative level of endogenous CKs in the gene-edited *GmCKX3* soybean. iPR, *N*^6^-(*∆*^2^ isopentenyl) adenosine; iP, *N*^6^-(*∆*^2^ isopentenyl) adenine; tZR, *trans*-zeatin riboside; tZ, *trans*-zeatin. Plants for the hormone analysis were grown in a field environment. Apical bud samples of 150 mg were collected at the flowering stage of the plants. Three independent replicates were analyzed for each event and represented as mean ± SD; all data were calculated using a Student’s *t*-test (*** *p* < 0.001, ** *p* < 0.002). (**b**) The subcellular localization of soybean GmCKX3-GFP fusion protein in tobacco leaves. *35s: GFP* is a *35s* promotor-driven *GFP* vector (the control). The expression of the ER localization gene *sper* used the *35s: SPER-mKATE* vector, showing a typical ER red signal fluorescence. Panels from left to right show the bright field, *GFP* signal channel, the *SPER-mKATE* signal channel, and the merged images, respectively. The expression of *35s: CKX3-GFP*, compared with *35s: SPER-mKATE*, also showed typical green fluorescence on the ER. Scale bar = 20 μm.

The RNA-Seq data from SoyOmics (https://ngdc.cncb.ac.cn/soyomics/index accessed on 12 December 2024) exhibited that *GmCKX3* in Williams82 expressed predominantly during the development of soybean leaf buds, flowers, and seed organs, especially with high expressions in flower-3, pod and seed-1, and seed-1 (Figure S2). To investigate whether the gene-edited *GmCKX3* affects the endogenous CK homeostasis, we quantified the content of CKs and derivatives, including iP, iPR, tZ, and tZR in the apical buds of the KO, and WT materials at the flowering stage, including leaf bud-3 and flower 3 via an LC–MS/MS (Figure 2a). As shown in Figure 2, compared to WT, the contents of iPR and iP in the KO mutants exhibited significant changes: the iPR content increased by 17–20%, while the iP content showed the greatest increase, nearly 60%. Interestingly, neither tZR nor tZ was significantly affected. Therefore, it can be speculated that the preferred substrates of *GmCKX3* are iP-type cytokinin and their nucleosides. Loss of function of *GmCKX3* loci resulted in remarkably increased cytokinin content in the apical buds of KO mutants.

To determine the subcellular localization of GmCKX3, the in-frame units of *35s: GmCKX3-GFP* were co-expressed with the ER-specific localization signal protein gene *sper* harboring the red fluorescent protein gene (*mkate* gene) in tobacco leaves. According to the confocal microscopy, the green fluorescence of the *GmCKX3-GFP* and the red fluorescence of the *SPER-mKATE* were merged onto the ER of tobacco cells (Figure 2b), revealing that GmCKX3 localizes specifically in the ER.

### 2.3. Gmckx3 Mutants Improved the Major Yield-Related Traits in Soybean

**Figure 3:**
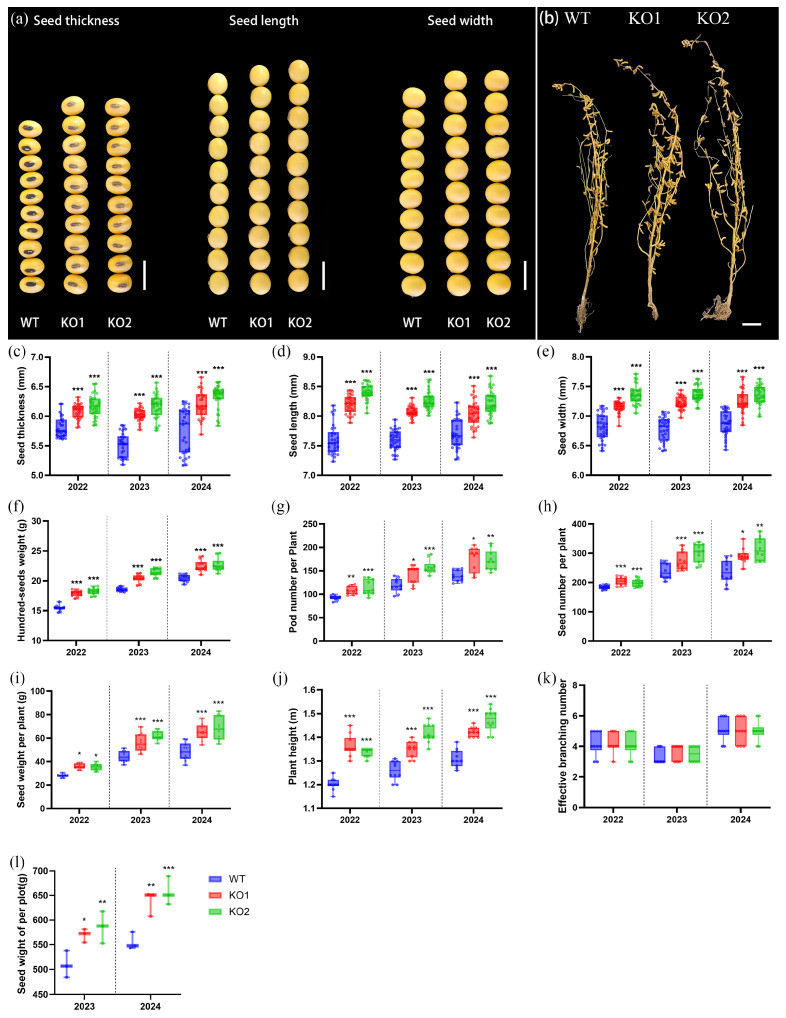
*Gmckx3* mutants improved the major yield-related traits in soybean. (**a**) The comparison of the seed thickness, seed length, and seed width of the gene-edited soybean. Scale bar = 1 cm. (**b**) The phenotype of the gene-edited soybean at the maturity stage. Scale bar = 10 cm. (**c**–**l**) The statistical analysis for three calendric years of KO mutants and WT. (**c**) Seed thickness (ST). (**d**) Seed length (SL). (**e**) Seed width (SW). (**f**) The 100-seed weight (100-SW). (**g**) Pod number per plant (PN). (**h**) Seed number per plant (SN). (**i**) Seed weight per plant (SWP). (**j**) Plant height (PH). (**k**) Effective branching number (EBN). (**l**) Seed weight of per plot (SW). One plot area of the field trial is 1 m^2^ in this study. All data are presented as means ± SD of 30 replicates for (**c**–**e**); of 3 replicates for (**l**); and of 10 replicates for others, respectively. All data were calculated using the Student’s *t* test (*** *p* < 0.001, ** *p* < 0.002, * *p* < 0.033).

In order to investigate the impact of *GmCKX3* on the yield-related traits in soybean, a comprehensive three-year phenotypic assessment was conducted using KO mutants (T2–T4 generations) and WT (Williams82). The yield-related traits involved plant height (PH), effective branching number (EBN), pod number per plant (PN), seed number per plant (SN), the 100-seed weight (100-SW), seed wight per plot (SWP), seed size (seed length (SL), seed width (SW), and seed thickness (ST)) in T2-T4 generations and plot yield in T3-T4 generations of KO mutants. As shown in Figure 3a,c–e, the KO mutants resulted in an increase in soybean SL, SW, and ST by 5% to 7%, 6% to 7%, and 8% to 10%, respectively. Similarly, the 100-seed weight increased by 12% to 15% in the KO mutants (Figure 3f). Interestingly, KO mutants showed significantly higher PN, SN, and SW than WT (Figure 3g–i). In the plot yield measurements for T3 and T4 generations of KO mutants, there was a significant increase in the yield, with increments ranging from 13% to 17% over their controls (Figure 3l). In addition, KO mutants gave rise to a greater plant height (Figure 3b,j), but no significant changes in the effective branch number (Figure 3k). Collectively, the knockout of *GmCKX3* has a diverse impact on yield-related traits in soybeans.

### 2.4. The Improvement of the Seed-Related Traits Is Likely Attributed to the Increase in the Cotyledon Cell Volume and Fat Content Caused by a GmCKX3 Deficiency in Soybeans

**Figure 4:**
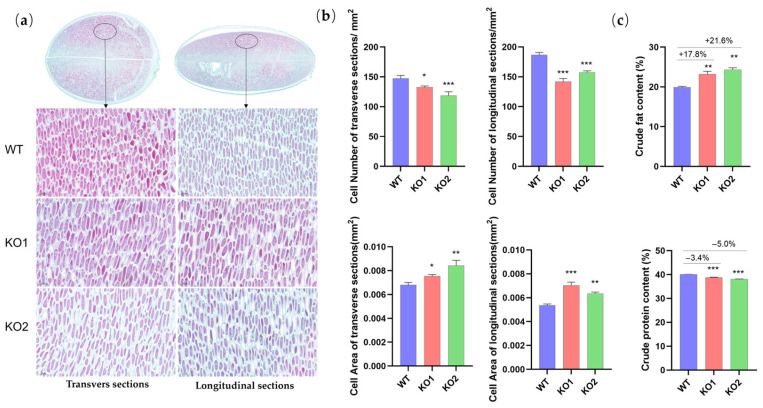
The improvement of the seed-related traits is likely attributed to the increase in the cotyledon cell volume and fat content caused by the *GmCKX3* deficiency in soybeans. (**a**) Paraffin-embedded sections of soybean seeds from the WT and KO mutants. Transverse sections are shown on the left, longitudinal sections on the right, with enlarged views of seed structures presented at the top. Scale bar = 100 μm. (**b**) The statistical results of the cell number and cell area in 1 mm^2^ of transverse and longitudinal sections. (**c**) The statistical results of the crude fat and protein content in mature seeds of KO mutants and WT. All data are presented as means ± SD of three replicates and calculated using the Student’s *t* test (*** *p* < 0.001, ** *p* < 0.002, * *p* < 0.033).

To understand the cause of the increase in seed-related traits in the genome-edited soybeans, we conducted a cellular dissection assessment to the mature seeds of the KO mutants and WT in both transverse (perpendicular to the seed hilum) and longitudinal (parallel to the seed hilum) sections, and the number of cotyledon cells per 1 mm^2^ area and the average individual cell cross-sectional area were measured using an image analysis software, Image-Pro Plus 6.0. The results in Figure 4a show that the KO seed cells in both the transverse and longitudinal sections appear to be larger than the WT. Logically, this led to a reduction in the unit-area cell number of both the transverse and longitudinal sections (Figure 4b). Meanwhile, in comparison with the WT, the average cell area of the KO seed cotyledon increased by 11% to 24% and 19% to 31% for transverse and longitudinal sections, respectively (Figure 4b).

During the cell expansion phase of cotyledon development, the biosynthesis and storage of fat and proteins represent fundamental processes [34,35]. A prevailing hypothesis posits that increased intracellular volume provides a greater capacity for fat-body accumulation [34]. However, the finite volume within the cell necessitates competition for space between protein bodies and fat bodies. Consequently, a well-established trade-off relationship exists between fat and protein production [35]. In addition, we assessed the cellular contents of soybean seed cotyledon and revealed that the crude fat content of the KO seeds was 18% to 22% higher than that of the WT seeds; conversely, the protein content decreased significantly by 3% to 5% (Figure 4c). These results indicated that the enlargement of the seed cotyledon cells, as well as the increase in the cellular fat content, are likely to be the critical causes contributing to the increase in the seed size and the seed weight, as observed in the phenotyping assessment of the *GmCKX3* edition soybean.

### 2.5. Transcriptomic Analysis of DEGs During Seed Development in the Gmckx3 Line

Our experiments continued to investigate the functional mechanism of *GmCKX3* on enhancing seed-related yield traits through catalyzing CKs in soybeans. A transcriptomic analysis was conducted on the pod and seed tissues at 2 weeks post-flowering (corresponding to the “pod & seed-1” in Figure S2) of WT and KO2 (*Gmckx3*-2). After confirmation of the data validation with PCA (Figure S3a), a total of 3943 DEGs were detected, including 2172 upregulated and 1771 downregulated genes (Figure S3b).

**Figure 5:**
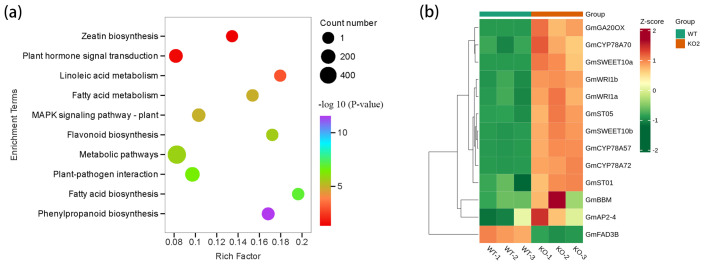
Transcriptomic analysis of DEGs during seed development in the gene-edited *GmCKX3* line. (**a**) Identification of enriched KEGG pathways in the comparison of WT and KO2 samples. The x- and y-axes indicate the enrichment factor and the metabolic pathway, respectively. The larger circles represent a greater number of enriched DEGs, and a redder coloration corresponds to a more significant enrichment. (**b**) A cluster heatmap of the reported genes related to the seed size and weight of soybeans in DEGs of RNA-seq.

In the KEGG analyses, these DEGs were enriched predominantly in zeatin biosynthesis, plant hormone signal transduction, phenylpropanoid biosynthesis and metabolism, linoleic acid metabolism, and fatty acid biosynthesis and metabolism (Figure 5a). However, the biological process and cellular function caused by the differential expression of *GmCKX3* were enriched in plant-type cell wall biogenesis, response to CK, response to gibberellin, fatty acid biosynthetic process, as well as the CK dehydrogenase activity in GO analyses (Figure S3e). Subsequently, seven DEGs involved in the zeatin biosynthesis pathway were subjected to qPCR analysis to validate the results of RNA-seq. It was found that the detected expression trends were consistent with those observed in RNA-Seq analyses for all DEGs tested (Figure S4). It confirms the reliability of these data and indicates that *GmCKX3* is one of the crucial performers involved in the zeatin biosynthesis pathway.

In these DEGs, a few important genes were reported to regulate the seed traits, such as several positive regulatory genes of *GmCYP78A57*, *GmCYP78A70,* and *CYP78A72* [36]; *GmST01* [37], *GmGA20OX* [38], *GmST05* [39], *GmSWEET10a,* and *GmSWEET10b* [40]; and one gene, *GmFAD3B,* with a negative regulatory effect [41] (Figure 5b). These genes regulated synergistically to enlarge the seeds of *Gmckx3* mutants.

### 2.6. Metabolomic Analysis of DAMs During Seed Development in the Gmckx3 Line

**Figure 6:**
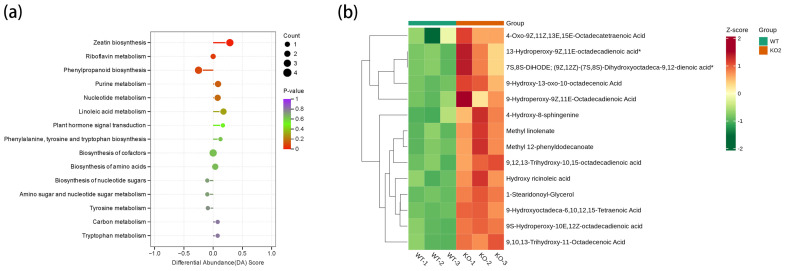
Metabolomic analysis of DAMs during seed development in the gene-edited *GmCKX3* line. (**a**) Enriched KEGG pathways of DAMs. The x- and y-axes represent the differential abundance score (DA Score) and the differential pathway names (sorted by *p*-value), respectively. (**b**) A cluster heatmap of lipid metabolites in the metabolome.

UPLC-MS/MS technologies were used in metabolomics analyses of WT and KO2 pod and seed-1. In line with the transcriptomic analyses, there were high levels of inter-group variability and intra-group reproducibility (Figure S3c). In total, 235 differentially abundant metabolites (DAMs) were detected in the Metware database, with 127 upregulated and 108 downregulated DAMs (Figure S3d). Coincidentally, the pathways enriched for the DAMs included zeatin biosynthesis, purine metabolism, linoleic acid metabolism, plant hormone signal transduction, etc., similar to those of the DEGs enrichment (Figure 6a).

Gene-edited *GmCKX3* altered the levels of 16 lipid metabolites (14 up) (Figure 6b), and the reported *GmSWEET10a* and *GmSWEET10b* genes with a positive regulatory effect on the fat content gene [40] were up-regulated in RNA-seq, which echoes the findings in Section 2.4 regarding the increased fat content.

### 2.7. Combinative Analyses of Transcriptome and Metabolome of Differential GmCKX3 Expression Revealed Important Genes and Pathways Involving CK Biosynthesis in Soybean

**Figure 7:**
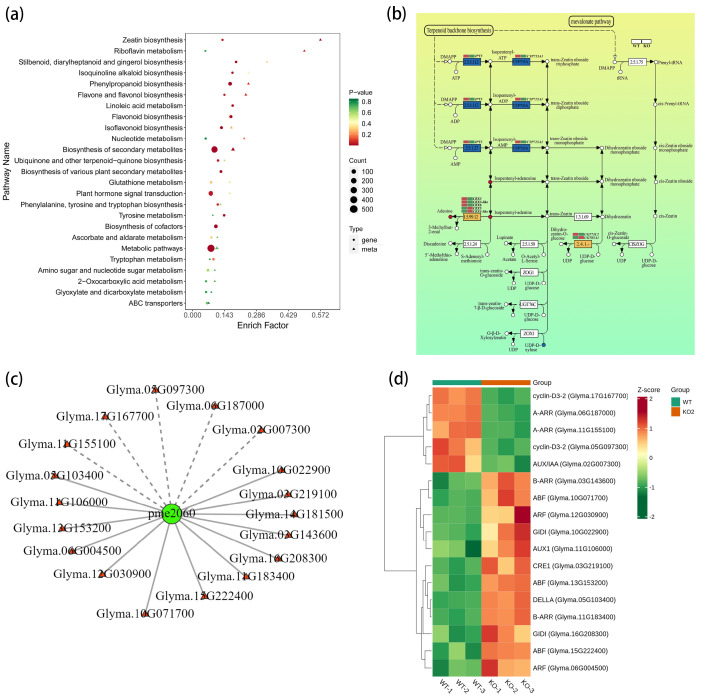
Combinative analyses of transcriptomes and metabolomes of differential *GmCKX3* expression revealed important genes and pathways involving CK biosynthesis in soybean. (**a**) Combined KEGG analysis of DEGs and DAMs. The x- and y-axes represent the enrichment factors and metabolic pathways, respectively. Circles and triangles indicate transcriptomic and metabolomic analyses, respectively. The shape size corresponds to the numbers of DEGs or DAMs, while red, yellow, and green colors indicate low, moderate, and high levels of enrichment, respectively. (**b**) The pathway of zeatin biosynthesis in transcriptomic and metabolomic analyses. The red and the dark blue dots indicate the upregulation of metabolites and detected but insignificant metabolites, respectively. The blue boxes represent downregulated DEGs, and the yellow boxes represent upregulated DEGs. The green small rectangles represent downregulation of expression relative to the red small rectangles. (**c**) Correlation network diagram of DAMs and DEGs in plant hormone signal transduction pathways. Related metabolites and genes are marked with green circle and red squares, respectively. The solid and the dashed lines represent positive and negative correlation network diagrams, respectively. (**d**) Cluster heatmap of DEGs in plant hormone signal transduction pathways of RNA-seq.

Coincidentally, the pathways enriched for the DAMs are similar to the DEGs enrichment, such as zeatin biosynthesis, purine metabolism, linoleic acid metabolism, and plant hormone signal transduction (Figure 7a). The combinative analysis of the DEGs and DAMs revealed a core gene cluster related to CK metabolism in the zeatin biosynthesis pathway (Figure 7b), including *CKX3* (*Glyma.17G054500*), *CKX3-like* (*Glyma.13G104700*), *CKX6* (*Glyma.12G011400*), *GmCKX5* (*Glyma.06G028900*), and *GmCKX1-like* (*Glyma.03G133300*). The expression patterns varied, with *CKX3*, *CKX3-like*, and *CKX6* upregulated, and *CKX5* and *CKX1-like* downregulated. Correlatively, upregulation was also observed in CKX3’s substrate *N*^6^-Isopentenyladenine (iP) and its precursor *N*^6^-isopentenyladenosine (IPR), as well as adenine, which is the product of a reaction catalyzed by CKXs. These results were consistent with the significant increase in iP-form CK in hormone content measurements.

The correlation network assay enriched *N*^6^-isopentenyladenine (iP, pme2060 in Figure 7c) as a core metabolite in plant hormone-signaling pathways. The iP was significantly upregulated, triggering the response factors of various hormones, such as CK receptor histidine kinase genes (*CRE1*), auxin influx carrier (*AUX1 LAX* family) genes, gibberellin receptor *GID1* gene, and ABA-responsive element binding factor (*ABF*) genes (Figure 7d).

Altogether, the gene editing of *GmCKX3* resulted in a significant accumulation of iP-form CKs. This suggests that functional *GmCKX3* plays a key role in the degradation of iP-form CKs. Elevated CK levels enhance cytokinin signaling and sensitivity to auxin and gibberellin pathways, collectively promoting endosperm cell division and elongation, as well as seed growth and development in the mutant [42,43,44,45]. Concurrently, while elevated CKs typically antagonize ABA signaling, the observed accumulation likely contributed to later seed maturation processes, where both hormones can play coordinated roles [43], and enhanced the seed traits of the *GmCKX3* mutant martials. All these findings are highly consistent with the morphological assessments in the genetically edited *GmCKX3* soybean in this study.

## 3. Discussion

Yield improvement has always been the core topic for soybeans, a key source of feeding protein and edible oil [5]. CKXs can regulate the endogenous level of CK to impact crop yield [8,25,46]. The present study characterized the functionality of the yield-related gene *GmCKX3*, derived from our previous GWAS, in soybean, using gene-editing approaches.

CKXs principally function in the biodegradation of CK derivatives; nevertheless, they can target diverse CK derivatives [47]. As reported previously, CKX3 mainly acts on the metabolism of iP and iPR in Arabidopsis [48], while it targets tZ and iP in rice shoots [49]. *OsCKX3* is responsible for the degradation of iP and cZ in roots under low NH4 + and of tZ, cZR, and iPR under high NH4+ [50]. In our study, *GmCKX3* was proven to be highly expressed during the early stages of flowering and seed development (Figure 1b). Genetic modification of *GmCKX3* selectively altered the content of active CK in WT, with the iP-form CK as the predominant active CK (Figure 4a). Therefore, *GmCKX3* in soybean, different from other plants, catalyzes preferentially the iP-form of CK as the substrate.

Over a continuous three-year field trial (Figure 3a,c–e), the gene-edited soybean exhibited average increases in SL, SW, and ST by 5–7%, 6–7%, and 8–10%, respectively. Similarly, Zhang et al. found that the variants of *TaCKX6-D1*, a wheat ortholog of *OsCKX2*, were strongly associated with the grain weight, showing an inverse correlation between *CKX* expression and thousand-grain weight across seven wheat varieties [28]. Meanwhile, Zalewski et al. demonstrated that an RNAi suppression of *HvCKX1* and *HvCKX9* s in barley increased the thousand-grain weight by 3%, the grain number by 6%, the spike number by 23%, and the final grain yield by 12% over the controls [30]. In cytological assessments of our study, we noticed that *GmCKX3* gene-edition in soybean led to a positive increase in cell volume than the unit-area of cell number (Figure 4), suggesting that *GmCKX3* knockout can promote cell expansion rather than accelerating cell division. Consistently, Liu et al. reported similar results in *CKX*-knockout cotton [51]. Perilli et al. also discovered that the inhibition of *CKX* expression accelerated cell expansion, during which the CK level played an important role [52]. In addition, our experiment also revealed increased PN and SN in the transgenic progenies (Figure 3g,h). Consistently, a study of Arabidopsis also suggested that the CK accumulation increased in the inflorescence meristem, leading to a yield increase of >20% [20]. Bartrina et al. documented that those elevated levels of CK in the inflorescences of a double *Atckx3ckx5* mutant produced enlarged inflorescences and an increased number of floral meristems, resulting in an augmented flower size, flower number, and silique number that ultimately gave rise to a 55% increase in the yield [26]. In contrast to the *CKX* function deficiency, *AtCKX3* overexpression could reduce the number of flowers in Arabidopsis, whereas *OsCKX2* overexpression reduced the grain numbers and the number of enlarged seeds in rice, attributable to a decreased primordium formation rate within the floral meristem [19,20].

Furthermore, according to the combinative analyses of the transcriptomes and metabolomes of the differential *GmCKX3* expression, there were divergent changes in CK metabolism-related gene expression and metabolite variations, providing new insights into the mechanism of *CKX*-mediated CK regulation. The transcriptomic analysis of *Gmckx3* mutants and WT plants during early seed development uncovered substantial alterations in five *GmCKXs* (downregulated: *GmCKX3*, *GmCKX3-like* and *GmCKX6*; upregulated: *CKX5* and *CKX1-like*) within the CK biosynthesis pathway (Figure 7b). Therefore, there exist potential compensatory and synergistic mechanisms among *CKX* genes, aligning with the conclusion that maintaining CK homeostasis requires coordinated action of multiple *GmCK*X*s* [16]. Notably, *GmCKX3* emerged as a key regulator of CK levels in reproductive organs such as flowers, pods, and seeds, with the absence of tissue-specific expression in other *CKX* genes so far [53]. Given this attribute, the upregulation of *CKX5* and *CKX1-like* failed to counterbalance the loss of *GmCKX3*, leading to reduced CK degradation as well as significantly accumulated iP, iPR, and adenine (Figure 2a and Figure 7b). Within the zeatin biosynthesis pathway, *IPT* (*Glyma.17G017400*) and *CYP735A* (*Glyma.09G137600*) were significantly downregulated, yet without the detection of major metabolite shifts, hinting a tissue-specific expression, as *Glyma.17G017400* is root-specific [54]. In addition, CK accumulation activated the GA receptor gene *GmGID1* and lipid biosynthesis gene *GmSWEET10a*, leading to an 18–22% lipid increase (Figure 4c), synergistically enhancing the seed filling. This trend may be achieved possibly through CK-GA co-signaling, similar to Arabidopsis *ARR1*-*GA20ox*-mediated elongation [55]. Contrasting root meristem antagonism [56], the upregulation of the auxin transporter gene AUX1 may promise CK-auxin synergy during seed expansion, implying tissue-specific hormone re-arrangement in *GmCKX3*-edited soybeans.

## 4. Materials and Methods

### 4.1. Construction of GmCKX3 Knockout Vectors

The *GmCKX3* knockout mutant (KO) was generated using a high-throughput genome-editing system [57]; and the vector was constructed using a dual sgRNA pool approach [58]. Two target sites (Target 1: 5′-TCCCACAGTGAATATCAAACGGG-3′; Target 2: 5′-TGACCCTGAAACCATTCAAATGG-3′.) were designed on the first exon of GmCKX3 using CRISPR-P2.0 [59]. The vectors used in this study (WMC015 and pUC57-ATMA) were modified and obtained from WiMi Biotechnology Co., Ltd. (Nanjing, China). The PCR fragments were amplified from pUC57-ATMA by the primers, p206112F:5′-CCAATGGTCTCATGATTGCCCACAGTGAATATCAAACGTTTTAGAGCTAGAAATAG-3′; p206112R:5′-ATTGGGGTCTCTAAACTTTGAATGGTTTCAGGGTCCAATCACTACTTCGACTCTAGC-3′.

### 4.2. Preparation of GmCKX3 Genetically Modified Mutants

The gene-edited vector p206112 was transformed into Williams 82 (W82) using the cotyledonary node method [60], giving rise to KO mutants. Sequencing validation was performed following the transfer of T1 plants into the soil and achieving robust growth. *Cas9* sequence fragments were detected via PCR in gene-edited T1 generation with the primers of Cas9-F: CACCATCTACCACCTGAGAA; Cas9-R: CGAAGTTGCTCTTGAAGTTG. Based on the positions of the dual-target sites designed within *GmCKX3*, a segment containing both targets was selected and amplified by PCR using the primers of J206112-F1: TCGGTCCGATATGTATACAGCC and J206112-R1: CCACCACAATCCCATCACGA.

For the field trial, the validated KO mutants were planted in rows in the field (spacing: 0.18 m). Gene-edited materials were screened from T1 generation plants for descendants without *Cas9* genes but with sequence deletion between the target sites.

### 4.3. Phenotyping of the Genetically Modified Soybean

The KO (T2–T4 generations) mutants and the WT were planted in the field of the Jilin Academy of Agricultural Sciences (E125°, N44°, China) for three seasoning years from 2022 to 2024. The KO (T2) and WT materials were planted in single-row fashion with a plant and row spacing of 0.18 m and 0.60 m, respectively. Then, KO (T3–T4) plants were cultivated in a typical soybean cultivation practice under a randomized complete block design with three replicates. Each plot consisted of four rows, with row lengths of 5 m, inter-plant distances of 0.18 m, and an inter-row spacing of 0.60 m. The plant growth and development were monitored and evaluated throughout the growth period. Upon maturity, 10 individual plants were randomly sampled from each replicate for subsequent assessment of the yield-related agronomic traits. The specific traits included the plant height (PH), the effective branch number (EBN), the number of pods per plant (PN), the number of seeds per plant (SN), the 100-seed weight (100-SW), the seed weight per plant (SWP), the seed weight per plot (SWPT) and the seed size traits of the seed length (SL), the seed width (SW), and the seed thickness (ST). Measurements and phenotypic evaluations were carried out based on specific protocols described previously [61,62,63].

### 4.4. Detection of Cytokinin Content

For cytokinin content detection, this experiment was initiated with the collection of the apical buds of the KO and WT materials at the flowering stage. Consequently, 15 plants were sampled independently and randomly divided into three groups, with 5 plants for each group serving as biological triplicates. Grounded powder (50 mg) was taken from each sample for detection. Cytokinin contents were detected using MetWare (http://www.metware.cn, accessed on 20 May 2024) based on the AB Sciex QTRAP 6500 LC-MS/MS platform.

### 4.5. Subcellular Localization of GmCKX3

Following the method described previously [64], the CDS sequence of *GmCKX3* was introduced into the expression vector pBWA (V) HS-X-GFP harboring the *CaMV35s* promoter (35s), i.e., *35s: GmCKX3-GFP*. An endoplasmic reticulum (ER)-specific marker signal protein gene *sper* was fused into the expression vector pBWA (V) HS-X-mKATE tagged with a red fluorescent protein gene *mkate* harboring the *CaMV35S* promoter (35s), i.e., *35s: SPER-mKATE*. The recombinant vectors harboring the expression frames of *35s:GmCKX3-GFP* and *35s: SPER-mKATE* were co-transformed into tobacco leaves. After two days of cultivation, the GFP (excitation: 488 nm, and emission: 510 nm) and mKATE (excitation: 561 nm, and emission: 580 nm) signals on the inoculated tobacco leaves were detected via laser confocal microscopy.

### 4.6. Paraffin Sections for Light Microscopy

Collect seeds from the KO mutants and WT R7 stages. Transverse (perpendicular to the seed hilum) and longitudinal (parallel to the seed hilum) sections were prepared using paraffin sections and stained with Safranin O and Fast Green following previously described methods [65]. The seed cells derived from the KO and WT within the paraffin sections were observed via tissue section digital scanner (Panoramic 250 FLASH, 3DHISTECH, Hungary). Three replications with a 10.0 × unit area field of view were examined, respectively, in the cross- and longitudinal sections of the seeds. The number of plant cells in each image field was counted, respectively, and the corresponding tissue area was measured using Image-Pro Plus 6.0 (Media Cybernetics, Baltimore, MD, USA) with mm as the standard unit. Then, the average cell area was calculated according to a formula where the average cell area equals the tissue area divided by the number of plant cells.

### 4.7. Measurement of the Crude Fat and Protein Content in Soybean Seeds

Quantification of crude fat and protein contents of the seeds was carried out at ProNet Biotech Co., Ltd. The T4 generation of *Gmckx3* mutants and WT mature seeds were used to measure the crude fat and protein content. The measurement method of the crude fat was a Soxhlet extraction method [66], and the crude protein content was determined using the Kjeldahl method [67].

### 4.8. RNA Extraction and Quantitative Real-Time PCR (qPCR) Analysis

Briefly, total RNA was extracted using the EasyPure**^®^** Plant RNA Kit (TransGen Biotech, Beijing, China), following the manufacturer’s protocol. Complementary DNA (cDNA) was synthesized using the TransScript**^®^** One-Step gDNA Removal and cDNA Synthesis SuperMix Kit (TransGen Biotech, Beijing, China). qPCR was performed with the TransStart**^®^** Tip Green qPCR SuperMix (+Dye I) reagent (TransGen Biotech, Beijing, China), with soybean *ACTIN11* (*Glyma.18G190800*) as the internal reference. Differential gene expression levels were calculated using the 2^−ΔΔCt^ method [68]. Specific primer sequences used for target gene amplification are summarized in Table S1.

### 4.9. Transcriptome Analysis

Total RNA was extracted from the pod and seed at the pod and seed-1 developmental stage (14 days after flowering) of both the KO mutant and WT, with an independent sampling of 15 plants and random division into three groups. RNA-seq was performed by Metware (Wuhan, China). Clean reads were sequentially compared with the Gmax_508_v4.0.fa.gz (https://data.jgi.doe.gov/refine-download/phytozome?q=Glycine+max+Wm82.a4.v1&expanded=Phytozome-508, accessed on 18 January 2024) using HISAT2 [69]. Quantitatively, the transcript expression levels were estimated from the fragments per kilobase of transcript per million fragments mapped [70]. Differentially expressed genes (DEGs) were identified based on a |log2 (fold change) | ≥ 1 and FDR < 0.05. Furthermore, the principal component analysis (PCA), heatmap, Gene Ontology (GO), and Kyoto Encyclopedia of Genes and Genomes (KEGG) enrichment analyses of DEGs were conducted using the Metware Cloud (https://cloud.metware.cn, accessed on 20 May 2024). The transcriptome datasets were deposited into the NCBI database (accession No.: PRJNA1218805).

### 4.10. Metabolome Profiling

The broad-targeted metabolomic analysis was performed by Metware (Wuhan, China). The samples for metabolome profiling were the same as those for the transcriptome analysis, with the detection of metabolites conducted via an ultra-performance liquid chromatography–electrospray ionization tandem mass spectrometry (UPLC-ESI-MS/MS). Metabolite quantification was performed using multiple reaction monitoring by triple quadrupole mass spectrometry [71]. Moreover, DAMs were identified based on fold change ≥2 and fold change ≤0.5. In addition, the PCA, heatmap, volcano plot, and KEGG enrichment of the DAMs were conducted with Metware Cloud (https://cloud.metware.cn, accessed on 20 May 2024). For the metabolome datasets, see Table S2.

### 4.11. Combinative Analysis of the Transcriptome and the Metabolome

The DEGs and DAMs of the KO and WT materials were mapped to the KEGG pathway diagram using the strategy described by Metware (Wuhan, China). Simultaneously, the heatmap and KEGG enrichment of the DEGs and DAMs, as well as the creation of a correlation network diagram, were conducted with Metware Cloud (https://cloud.metware.cn, accessed on 20 May 2024).

### 4.12. Statistical Analysis

All experimental data were collected from a minimum of three independent replicates and analyzed statistically using GRAPHPAD PRISM 10 (GraphPad Software 10, San Diego, CA, USA).

## 5. Conclusions

This study demonstrates that the CRISPR/Cas9-mediated knockout of *GmCKX3* significantly enhances soybean seed yield by disrupting cytokinin homeostasis. *GmCKX3* preferentially degrades isopentenyladenine (iP)-type cytokinins, and its functional loss elevates the endogenous iP levels by >60%, triggering cotyledon cell expansion rather than proliferation, thereby directly enlarging the seed size. Concurrently, elevated cytokinin signaling upregulates lipid biosynthesis pathways, increasing the seed oil content by 18–22% despite a marginal protein reduction. Integrated transcriptomic–metabolomic analyses further reveal synergistic activation of auxin, gibberellin, and ABA signaling, collectively promoting seed filling. These findings establish *GmCKX3* as a key regulator of yield traits and provide a strategic target for engineering high-yield, high-oil soybean cultivars through cytokinin pathway modulation.

## Data Availability

The original contributions presented in this study are included in the article/Supplementary Materials. Further inquiries can be directed to the corresponding authors.

## Supplementary Materials

The following supporting information can be downloaded at https://www.mdpi.com/article/10.3390/plants14142207/s1; Figure S1: The gene-editing and overexpression vector structure and expression validation of *GmCKX3*; Figure S2: Tissue-specific expression profile of *GmCKX3* in soybean Williams82; Figure S3: Bioinformatics Analysis of DEGs and DAMs; Figure S4: The expression levels of 7 DEGs of zeatin biosynthesis pathway were verified by qRT-PCR; Table S1: Specific primer sequences used for target gene amplification; Table S2: metabolome datasets.
