# Supplementary material for: Knockout of GmCKX3 Enhances Soybean Seed Yield via Cytokinin-Mediated Cell Expansion and Lipid Accumulation
Source: Plants (Basel). 2025 Jul 16;14(14):2207. doi: 10.3390/plants14142207 (PMC12299880; doi:10.3390/plants14142207)

Figure S1. The gene-editing and overexpression vector structure and expression validation of *GmCKX3*

The gene-editing expression vector structure of *GmCKX3*.

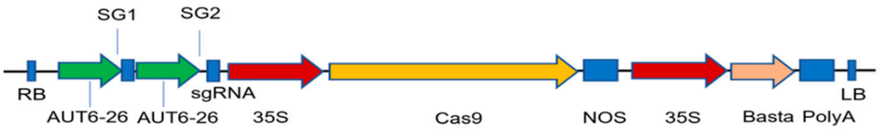

Figure S2. Tissue-specific expression profile of *GmCKX3* in soybean Williams82.

Tissue-specific expression profile of *GmCKX3* in soybean Williams82 from the SoyOmics (https://ngdc.cncb.ac.cn/soyomics/index). The deeper red color indicates a higher relative expression level of *GmCKX3* in the tissue.

Glyma.17g054500

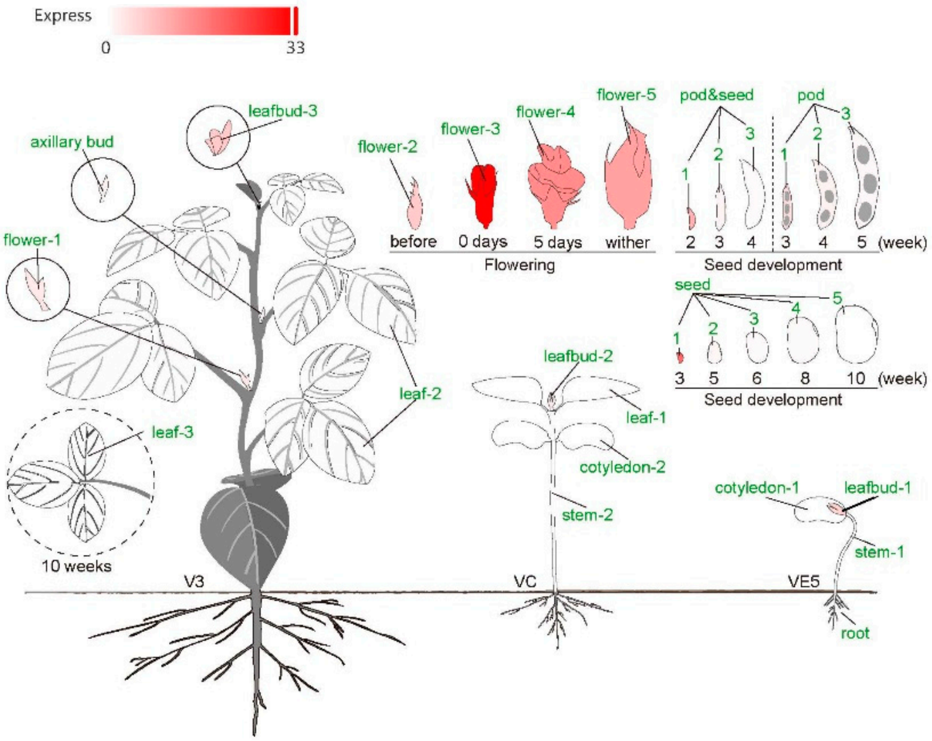

Figure S3. Bioinformatics Analysis of DEGs and DAMs

(a) The PCA analysis of transcriptome. The x-axis represents PC1, the y-axis represents PC2, and the dots of different colors represent samples in different groups. **(b)** Volcano map of DEGs based on the transcriptome analysis of WT and KO2 samples. The x-axis indicates the change of gene expression multiple, and the y-axis indicates the significance level of differential genes. The red dots represent up-regulated differential genes, the green dots represent down-regulated differential genes, and the gray dots represent non-differentially expressed genes. **(c)** The PCA analysis of metabolome. **(d)** Volcano map of DAMs based on the metabolome analysis of WT and KO2 samples. **(e)** Enriched gene ontology pathways of the DEGs. y-axis represents biological functions; different colors indicate different categories; x-axis represents the percentage of genes enriched in this pathway among all genes from RNA-seq.

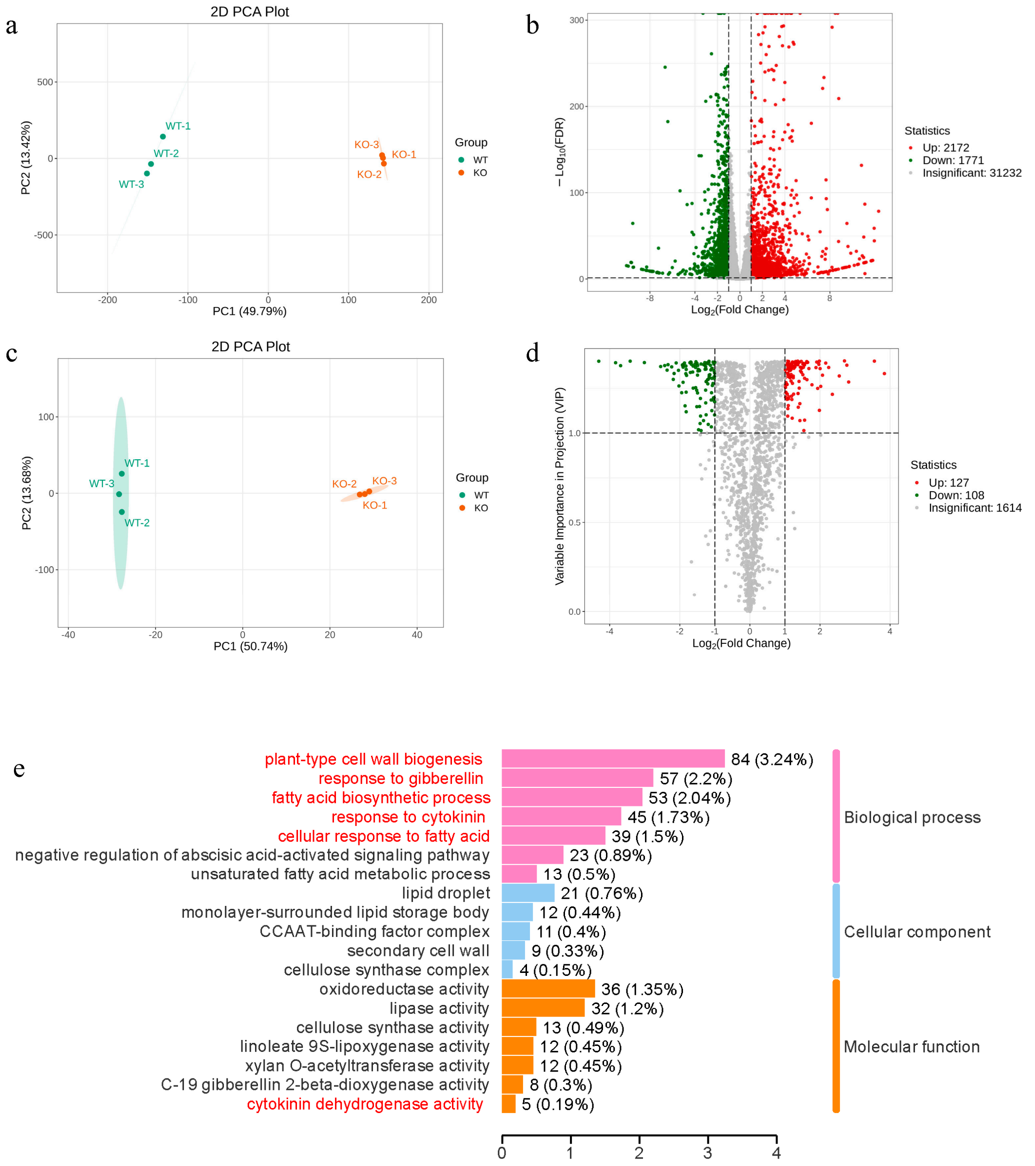

Figure S4. The expression levels of 7 DEGs of zeatin biosynthesis pathway were verified by qRT-PCR. The left two bars correspond to RNA-seq data, while the right two bars correspond to qRT-PCR data in KO1 and KO2.

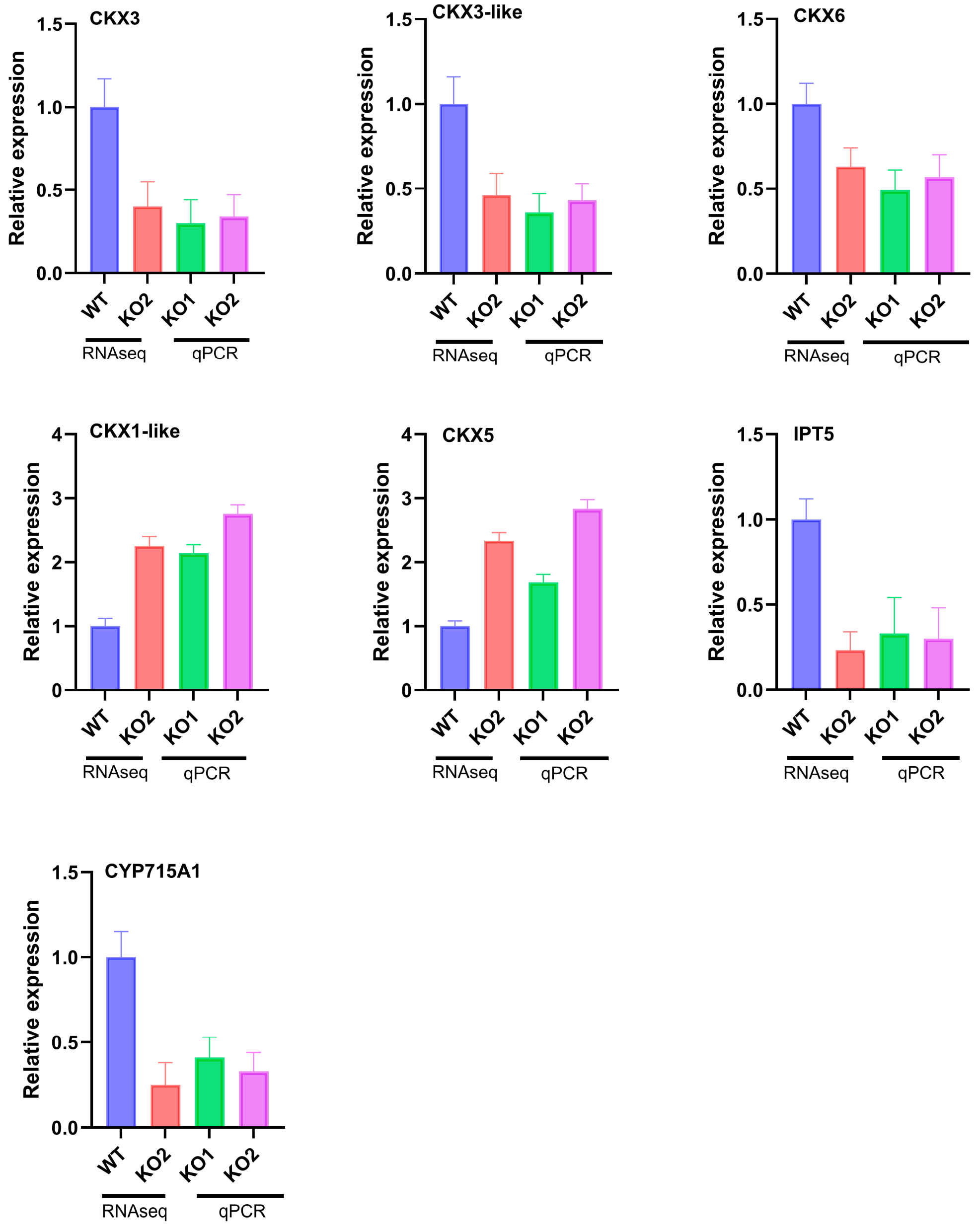

Supplement: Supplementary file 1 [file plants-14-02207-s001.zip › Supplementary figures.pdf]
