# Supplementary material for: Knockout of GmCKX3 Enhances Soybean Seed Yield via Cytokinin-Mediated Cell Expansion and Lipid Accumulation
Source: Plants (Basel). 2025 Jul 16;14(14):2207. doi: 10.3390/plants14142207 (PMC12299880; doi:10.3390/plants14142207)
Supplement: Supplementary file 1 [file plants-14-02207-s001.zip › Supplementary Table S1.pdf]

Table S1 Specific primer sequences used for target gene amplification

Primer Name	Sequence(5'to3')
Actin11-F	TGCCCTCCCACATGCCATCCT
Actin11-R	TCGGCTGAGGTGGTGAAGGAATAAC
CKX3-F	ATGGCTCTAAACTACCCT
CKX3-R	ACACTAATCGCAACCC
IPT5-F	CACCACGAACAAAGTCAC
IPT5-R	CCAGAGGAAGCAGCAC
CKX6-F	ATGAGATACCATTACCCACC
CKX6-R	CCTGTTGCCGAAGTCC
CKX3-like-F	ATAGCAAGGGCGAGAA
CKX3-like-R	CAGATAGAGGGAAGAAAGAG
CYP735A-F	TAGGGTCCTCTAATCTCAC
CYP735A-R	TTCTGTACCAAGCCAATA
CKX1-like-F	CTGGCATAAGTGGGC
CKX1-like-R	AAGGTCAGCGTTTCG
CKX5-F	GGGGTGGGAAGTTGT
CKX5-R	TGAGGACCGTGATTGA
